# A clotting time longer than 226 s in the INTEM channel of the thromboelastometer is an independent risk factor for mortality during bleeding

**DOI:** 10.1007/s00101-025-01602-w

**Published:** 2025-11-10

**Authors:** Hagen Bomberg, Klaus Görlinger, Stefan Wagenpfeil, Thomas Volk, Sven Oliver Schneider

**Affiliations:** 1 Department of Anaesthesiology, Intensive Care Medicine and Pain Medicine, Saarland University, University Medical Centre, Homburg/Saar, Germany; 2 Department of Anaesthesiology, Intensive Care Medicine and Pain Medicine, Balgrist University Hospital, University of Zurich, Forchstrasse 370, 8008 Zürich, Switzerland; 3 Department of Outcomes Research Consortium, UTHealth, Houston, TX USA; 4 Medical Director, Tem Innovations GmbH, Munich, Germany; 5 Institute for Medical Biometry, Epidemiology and Medical Informatics, Saarland University, University Medical Centre, Homburg/Saar, Germany

**Keywords:** Mortality, Risk factor, Bleeding, Rotational thromboelastometry, Coagulation, Mortalität, Risikofaktor, Hämorrhagie, Rotationsthrombelastometrie, Gerinnung

## Abstract

**Background and objective:**

During bleeding the prolongation of the clotting time (CT_INTEM_) measured by rotational thromboelastometry (ROTEM) can detect alterations in the intrinsic pathway; however, the significance of a prolonged CT_INTEM_ for risk stratification in patients with bleeding and the treatment with fresh frozen plasma remains unclear.

**Material and methods:**

A total of 2197 consecutive patients between 2014 and 2020 were retrospectively investigated. All patients were tested by ROTEM during bleeding at the Saarland University Hospital. The CT_INTEM_ values were compared to mortality at 30 days. Discrimination was assessed with C statistic. Adjusted hazard ratios (adjHR, 95% confidence interval, CI) were calculated with multivariable Cox models.

**Results:**

The results of the C‑statistic showed that CT_INTEM_ (C statistic 0.62, optimal threshold > 226 s) had a predictive power for 30-day mortality. The determined threshold value of CT_INTEM_ > 226 s remained an independent risk predictor for 30-day mortality even after adjustment for confounding factors (adjHR 2.6, 95% CI 2.1–3.2, *p* < 0.001). The 30-day mortality rate was significantly increased in the group with CT_INTEM_ > 226 s (29% versus 15%, *p* < 0.001). A multivariable analysis showed that treatment with fresh frozen plasma was not associated with increased 30-day mortality in patients with CT_INTEM_ > 226 s, in contrast to all patients.

**Conclusion:**

Our results indicate that CT_INTEM_ > 226s detected alterations in the intrinsic pathway might be an independent predictor for 30-day mortality in patients with bleeding and could be useful for decision making regarding treatment with fresh frozen plasma.

**Supplementary Information:**

The online version of this article (10.1007/s00101-025-01602-w) contains supplementary material, which is available to authorized users.

## Abstract (de)

**Hintergrund:**

Bei Blutungen kann die Verlängerung der CT_INTEM_ („clotting time“), gemessen mit der Rotationsthrombelastometrie (ROTEM), Veränderungen des intrinsischen Gerinnungswegs aufzeigen. Die Bedeutung einer verlängerten CT_INTEM_ für die Risikostratifizierung bei Patienten mit Blutungen und die Therapie mit gefrorenem Frischplasma ist immer noch unklar.

**Material und Methoden:**

Zwischen 2014 und 2020 wurden 2197 konsekutive Patienten retrospektiv untersucht. Alle Patienten wurden mit Blutungen am Universitätsklinikum des Saarlandes mittels ROTEM getestet. Die CT_INTEM_-Werte wurden mit der 30-Tages-Sterblichkeit verglichen. Die Diskriminierung wurde mittels C‑Statistik bewertet. Angepasste Hazard-Ratios (adjHR [95 % Konfidenzintervall]) wurden mittels multivariabler Cox-Modelle berechnet.

**Ergebnisse:**

Die C‑Statistik zeigte, dass CT_INTEM_ (C-Statistik = 0,62, optimaler Schwellenwert > 226 s) prädiktiv für die 30-Tage-Sterblichkeit war. Der festgelegte Schwellenwert von CT_INTEM_ > 226 s blieb auch nach Anpassung an Störfaktoren ein unabhängiger Risikoparameter für die 30-Tages-Sterblichkeit (adjHR 2,6 [2,1–3,2]; *p* < 0,001). Die 30-Tages-Sterblichkeit war in der Gruppe mit CT_INTEM_ > 226 s deutlich erhöht (29 % vs. 15 %; *p* < 0,001). Eine multivariate Analyse zeigte, dass die Behandlung mit gefrorenem Frischplasma bei Patienten mit CT_INTEM_ > 226 s nicht mit einer erhöhten 30-Tages-Sterblichkeit assoziiert war, im Gegensatz zur Gesamtkohorte.

**Schlussfolgerung:**

Eine CT_INTEM_ > 226 s zeigt eine Veränderung im intrinsischen Gerinnungsweg und scheint ein unabhängiger Risikofaktor für die 30-Tages-Sterblichkeit bei Patienten mit Blutungen zu sein. Dies könnte in der Entscheidungsfindung zur Therapie mit gefrorenem Frischplasma nützlich sein.

## Introduction

Bleeding can result in complex coagulation disorders and hemostatic failure. Rotational thromboelastometry (ROTEM) is a point-of-care method using whole blood samples for the diagnosis of hemostatic disorders [1, 2].

All ROTEM analyses could be performed with single use reagents consisting of three tissue factor-activated assays, EXTEM (extrinsic pathway), FIBTEM (fibrin contribution to clot firmness), APTEM (inhibition of fibrinolysis) and two contact-activated assays INTEM (intrinsic pathway without heparin neutralization) and HEPTEM (intrinsic pathway with heparin neutralization). All assays estimate clotting time (CT), clot formation time (CFT), clot firmness and fibrinolytic processes [3].

Prolongation of clotting time in INTEM (CT_INTEM_) in trauma patients was significantly associated with death [4, 5]. A CT_INTEM_ over 240 s was found in 11/22 nonsurvivors after severe traumatic brain injury in an analysis by Schöchl et al. [5]. A therapeutic approach for patients with CT_INTEM_ over 240 s is the administration of fresh frozen plasma [2, 6–8].

Our aim was therefore to investigate the relationship between CT_INTEM_, 30-day mortality and fresh frozen plasma treatment. Our primary hypothesis was that there is an independent association between CT_INTEM_ and 30-day mortality in patients with bleeding. Second, we hypothesized that fresh frozen plasma might be associated with 30-day mortality in patients with elevated CT_INTEM_.

## Material and methods

The study was registered in German Clinical Trials registration (registration number DRKS00026153) on September 2021 (principal investigator: PD Dr. med. Sven Oliver Schneider, Saarland University, Department of Anesthesiology, Intensive Care Medicine and Pain Medicine, Homburg/Saar, Germany). After approval by the ethics committee (Ärztekammer Saarland; number 93/20), the data were retrospectively collected. The requirement for written informed consent was waived by the ethics committee.

### Inclusion and exclusion criteria

All patients tested in the Saarland University Hospital by ROTEM between 2014 to 2020 were included and is available for all patients with bleeding and/or coagulation disorders in the Saarland University Hospital. Bleeding is defined as patients suffering from blood loss determined at the discretion of the treating physician. Exclusion criteria for the study population were missing information about CT_INTEM_ measurement, implausible data and missing information on the variables listed in Tables 1 and 2.

**Table 1:** Population characteristics and comorbidities of patients before rotational thromboelastometry (ROTEM) analysis

	*CT*_*INTEM*_* ≤* *226* *s*		*CT*_*INTEM*_* >* *226* *s*		*p‑value*
	*(n* *=* *1496)*		*(n* *=* *701)*		
*Age (years)*	63	±17	62	±17	*0.1*
*Male (%)*	942	(63)	465	(66)	*0.1*
*Body mass index (kg/m*^*2*^*)*	27	±6	27	±6	*0.4*
*Emergency*	492	(33)	201	(29)	**0.05**
***Department***					
*General surgery (without liver)*	414	(28)	124	(18)	**<** **0.001**
*Liver surgery*	126	(8)	49	(7)	*0.3*
*Cardiothoracic and vascular surgery with CPB*	414	(28)	409	(58)	**<** **0.001**
*Traumatology and orthopedic surgery*	276	(18)	38	(5)	**<** **0.001**
*Cardiology and pulmonology*	327	(22)	181	(26)	**0.05**
*Other departments*	422	(28)	152	(22)	**0.001**
*Reoperation after postoperative bleeding*	346	(23)	165	(24)	*0.8*
***Comorbidities***					
*Coronary heart disease*	375	(25)	223	(32)	**0.001**
*Cardiac insufficiency (EF* *<* *45%)*	*98*	(7)	59	(8)	*0.1*
*Chronic obstructive pulmonary disease*	142	(9)	60	(9)	*0.5*
*Insulin-dependent diabetes mellitus*	103	(7)	48	(7)	*1*
*Liver disease child C*	62	(4)	41	(6)	*0.1*
*Coagulation disorders*	179	(12)	81	(12)	*0.8*
***Anticoagulation prior to ROTEM analysis***					
*Aspirin 100* *mg*	333	(22)	198	(28)	**0.003**
*High molecular weight heparin*	248	(17)	88	(13)	**0.02**
*Low molecular weight heparin*	177	(12)	30	(4)	**<** **0.001**
*Other anticoagulation*	88	(6)	72	(10)	**<** **0.001**

Continuous variables are expressed as mean and standard deviation. Categorical variables are presented as numbers (column percentages) and were compared with χ^2^ tests. Emergency at hospital admission is defined as surgical intervention necessary within 6 h after hospital admission. Other departments: pediatric, eye, anesthesia. thoracic surgery without CPB, vascular surgery without CPB, gynecology, obstetrics, urology, neurosurgery, ear, nose, throat and maxillofacial surgery. Coagulation disorders: known bleeding coagulation disorder, e.g. thrombocytopenia, factor deficiency, von Willebrand disease. Anticoagulation prior to the ROTEM analysis includes anticoagulation on the hospital ward or at home. Other anticoagulation: argatroban, phenprocoumon, factor Xa inhibitor, dabigatran and P2Y12 inhibitors. Traumatology and orthopedic surgery also include multiple injury/polytraumatized patients

*CT*_*INTEM*_ clotting time in seconds (s), *CPB* cardiopulmonary bypass, *EF* ejection fraction, *ROTEM* rotational thromboelastometry

**Table 2:** Outcome related to CT_INTEM_ (clotting time) in seconds (s)

	Before ROTEM analysis					After ROTEM analysis				
	*CT*_*INTEM*_* ≤* *226s*		CT_INTEM_ > 226s		*p*-value	CT_INTEM_ ≤ 226s		CT_INTEM_ > 226s		*p*-value
	(*n* = 1496)		(*n* = 701)			(*n* = 1496)		(*n* = 701)		
*Mechanically ventilated (h)*	18	±96	33	±165	**0.007**	60	±166	67	±178	*0.4*
*Renal replacement therapy (%)*	141	(9)	130	(19)	**<** **0.001**	233	(16)	213	(30)	**<** **0.001**
***Length of stay (days)***										
*In ICU*	3	±9	3	±10	**0.04**	9	±16	11	±21	**0.05**
*In hospital*	7	±13	8	±17	**0.03**	23	±32	19	±30	*0.1*
*30-day mortality (%)*	–		–		–	224	(15)	200	(29)	**<** **0.001**
*Packed red blood cells (n)*	2	±5	3	±7	**0.001**	4	±5	4	±6	*0.2*
*Platelet concentrate (n)*	0.8	±2.1	1.2	±3.3	**<** **0.001**	1	±2	2	±2	**<** **0.001**
*Fresh frozen plasma (n)*	0.3	±1.8	0.9	±6	**0.001**	1	±20	2	±9	*0.2*
*Prothrombin concentrate (IU)*	390	±1480	1042	±5464	**<** **0.001**	383	±1411	938	±1819	**<** **0.001**
*Fibrinogen (g)*	0.5	±2.3	1.1	±5.3	**<** **0.001**	0.6	±2	1.1	±3	**<** **0.001**
*Recombinant factor VIIa (IU)*	1.7	±40	4.4	±61	*0.2*	3	±45	13	±151	**0.03**
*Antithrombin III (IU)*	98	±671	248	±1514	**0.001**	72	±708	112	±915	*0.3*
*Factor XIII (IU)*	31	±359	41	±439	*0.6*	73	±1090	34	±234	*0.4*
***Adverse events***										
*Pneumonia (%)*	129	(9)	69	(10)	*0.4*	131	(9)	98	(14)	**<** **0.001**
*Pulmonary embolism (%)*	31	(2)	15	(2)	*1*	13	(1)	7	(1)	*0.8*
*Acute myocardial infarction (%)*	55	(4)	37	(5)	*0.1*	9	(1)	5	(1)	*0.8*
*Embolic apoplexy (%)*	37	(2)	33	(5)	**0.009**	16	(1)	21	(3)	**0.002**
*Peripheral arterial embolism and thrombosis (%)*	44	(3)	32	(5)	*0.1*	6	(0.4)	9	(1)	**0.03**
*Gastrointestinal ischemia (%)*	40	(3)	46	(7)	**<** **0.001**	28	(2)	35	(5)	**<** **0.001**
*Gastrointestinal bleeding (%)*	101	(7)	33	(5)	*0.1*	12	(1)	8	(1)	*0.5*

The outcome was split into the time before and after the ROTEM analysis. Continuous variables are expressed as mean and standard deviation. Categorical variables are presented as numbers (percentages)

*ICU* intensive care unit, *ROTEM* rotational thromboelastometry. Packed red blood cells (450 ml), platelet concentrate (270 ml), fresh frozen plasma (300 ml)

### Data source

Rotational thromboelastometry data are recorded during patient care and stored in the hospital’s medical reports and the bleeding database. These include patients with and without surgery. Detailed information about the medical conditions of patients along with the procedure were extracted from the hospital database (Tables 1 and 2).

### Rotational thromboelastometry analyses

The ROTEM (ROTEM delta®, Tem Innovations, Munich, Germany) analyses in this study include only measurements of two contact-activated assays (INTEM intrinsic pathway without heparin neutralization). All assays estimate clotting time (CT). Each patient was considered only once. If more than one ROTEM analysis was done in one patient, only the first analysis was considered.

### Proof of plausibility

Data integrity was evaluated according to specific rules to delete erroneously entered data and cases with missing information (proof of plausibility, Appendix 1).

### Missing data handling/sensitivity analyses

Patients with missing data were excluded from the analysis. Several sensitivity analyses were performed.

### Definition of the outcomes

The primary outcome was overall survival with follow-up period of 30 days.

The secondary outcomes were severe adverse events defined by the occurrence of pneumonia, pulmonary embolism, acute myocardial infarction, embolic apoplexy, peripheral arterial embolism and thrombosis, gastrointestinal ischemia (measured by angiography, computed tomography or intraoperative finding) and gastrointestinal bleeding.

### Data analysis

Receiver operating characteristic curves were constructed (C statistic) to evaluate the predictive power of CT_INTEM_ for the occurrence of 30-day mortality. The Youden Index was used to calculate the optimal threshold for CT_INTEM_ in prediction of 30-day mortality.

Continuous variables are expressed as means ± standard deviations and were compared using Student’s t‑test. Categorical variables are presented as absolute and relative frequencies and were compared with χ^2^ tests.

The number needed to screen was calculated to measure how many patients must be screened with values CT_INTEM_ > 226s to avoid 1 death. The positive predictive value was defined as number of true positives/(number of true positives + number of false positives). The negative predictive value was defined as number of true negatives/(number of true negatives + number of false negatives).

Survival rates were estimated using the Kaplan-Meier method and compared using the log-rank test. Cox proportional hazards models were used to adjust for confounding factors including all variables from Table 1 with *p* ≤ 0.05. Pairwise dependent variable constellations with Pearson or Spearman correlation coefficients exceeding +0.4 or less than −0.4 were a priori specified as interaction terms in multivariable analyses to account for the issue of multicollinearity.

Sensitivity analyses were performed including confounding factors listed in Tables 1 and 2. In an additional sensitivity analysis, the data were randomly split into two groups based on the month they underwent ROTEM analysis in an alternating manner. To avoid any bias associated with the month of ROTEM analysis, we started each year with another group, also alternating.

Data analysis was performed using SPSS Statistics 19™ (IBM, Ehningen, Germany) and two-sided *p* values ≤ 0.05 were considered statistically significant.

## Results

During the study period 2656 patients had documented index ROTEM analyses. Among these 459 patients did not meet the inclusion criteria by virtue of missing information about CT_INTEM_ analysis, comorbidities or outcome. The final study population includes 2197 patients.

**Fig. 1:**
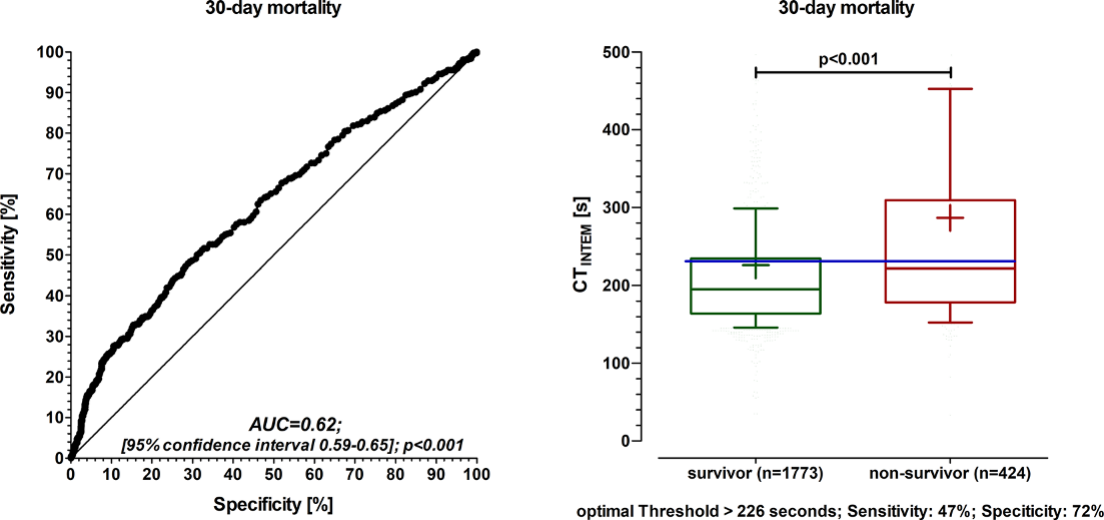
CT_INTEM_ (clotting time) and ROC analysis for mortality (*left graph*). Box and whisker plot with the 10th and 90th percentiles (*right graph*). *Solid line* in the box: median values of CT_INTEM_. The *plus symbol* in the box: mean values of CT_INTEM_. *Blue line*: optimal threshold for CT_INTEM_ in prediction of 30-day mortality. Receiver operating characteristic curves (ROC) were constructed (C-statistic) to evaluate the predictive power of CT_INTEM_ for 30-day mortality. *AUC* area-under-the-curve. The Youden Index was used to calculate optimal threshold for CT_INTEM_ in prediction of 30-day mortality. Data were collected in real-time by the attending physician or nurse in parallel to the patient treatment. Each patient was considered only once. If more than one Rotem (rotational thromboelastometry) analysis was done in one patient, only the first Rotem analysis was considered

The results of the C‑statistic showed that CT_INTEM_ had a discrimination for prediction of 30-day mortality (optimal threshold > 226 s, C‑statistic 0.62, sensitivity 47% and specificity 72%; Fig. 1). The number needed to screen (NNS), the positive predictive value (PPV), and the negative predictive value (NPV) were calculated for 30-day mortality using the optimal threshold of CT_INTEM_ > 226 s (NNS: 7, PPV: 0.29, NPV: 0.85).

Patients with CT_INTEM_ > 226s had less orthopedic or general surgery and more surgery with cardiopulmonary bypass (Table 1). Interestingly, the comorbidities during hospital admission were not significantly different except for coronary heart disease. The outcome was split into the time before and after the ROTEM analysis. Patients with CT_INTEM_ > 226s required more blood transfusions, had more renal replacement therapy and required more coagulation factors (Table 2).

**Fig. 2:**
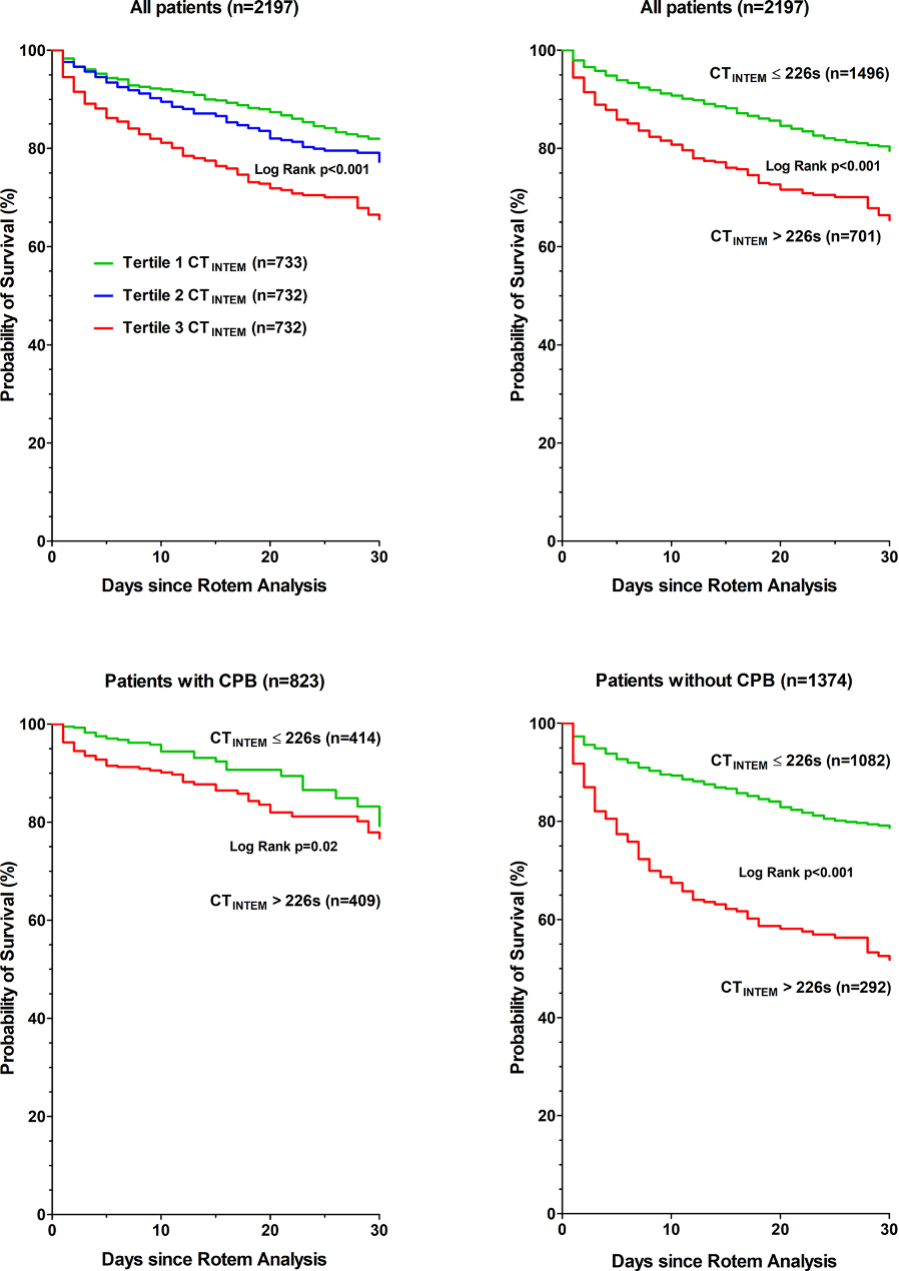
All patients (*n* = 2197) and subgroups analyses in patients with (*n* = 823) or without (*n* = 1374) cardiopulmonary bypass. Kaplan-Meier survival plots for terciles of clotting time (CT_INTEM_), CT_INTEM_ > 226 s versus CT_INTEM_ ≤ 226s for 30 days follow-up. *ROTEM* rotational thromboelastometry, *CPB* cardiopulmonary bypass

Patients with CT_INTEM_ > 226s had an increased 30-day mortality compared to patients with CT_INTEM_ ≤ 226s (29% versus 15%, *p* < 0.001; Table 2; Fig. 2). Even after adjustment for confounding factors CT_INTEM_ > 226s remained an independent risk factor for 30-day mortality. This remained true in sensitivity analyses (Table 3a). We compared survival versus nonsurvival in patients suffering from CT_INTEM_ over 226 s. Acute myocardial infarction, gastrointestinal bleeding, gastrointestinal ischemia, renal replacement therapy and therapy with prothrombin concentrate and fibrinogen were seen significantly more often in nonsurvivors (Appendix 2).

**Table 3:** A: Primary model, sensitivity and subgroup analyses. B: Does fresh frozen plasma have an impact on 30-day mortality after a ROTEM analysis?

**Table 3A Cox proportional hazard models: CT**_**INTEM**_** >226s a risk factor for 30-day mortality**		
***Model***	***HR (95% CI)***	***p‑value***
**Crude in all patients (*n*** **=** **2197)**	2.1 (1.8–2.6)	**<** **0.001**
**Primary model in all patients (*n*** **=** **2197)**		
*Adjusted all variables from table 1 with p* ≤ *0.05*	2.6 (2.1–3.2)	**<** **0.001**
**Sensitivity analyses in all patients (*n*** **=** **2197)**		
*Adjusted all variables from table 1*	2.7 (2.2–3.3)	**<** **0.001**
*Adjusted all variables from table 1 and all variables before ROTEM analysis from table 2*	2.0 (1.6–2.5)	**<** **0.001**
**Sensitivity analyses in randomly split group 1**^a^ (***n*** **=** **1106)**		
*Crude*	2.5 (1.9–3.2)	**<** **0.001**
*Adjusted all variables from table 1 with p* *≤* *0.05*	3.1 (2.3–4.1)	**<** **0.001**
*Adjusted all variables from table 1 and all variables before ROTEM analysis from table 2*	2.5 (1.8–3.5)	**<** **0.001**
**Sensitivity analyses in randomly split group 2**^a^ (***n*** **=** **1091)**		
*Crude*	1.9 (1.4–2.4)	**<** **0.001**
*Adjusted all variables from table 1 with p* *≤* *0.05*	2.3 (1.7–3.0)	**<** **0.001**
*Adjusted all variables from table 1 and all variables before ROTEM analysis from table 2*	1.6 (1.2–2.2)	**0.004**
**Subgroup analyses in patients who underwent cardiothoracic and vascular surgery with cardiopulmonary bypass (*n*** **=** **823)**		
*Crude*	1.8 (1.2–2.7)	**0.007**
*Adjusted all variables from table 1 with p* *≤* *0.05*	1.8 (1.2–2.7)	**0.008**
*Adjusted all variables from table 1 and all variables before ROTEM analysis from table 2*	1.5 (0.9–2.4)	*0.1*
**Subgroup analyses in patients without cardiopulmonary bypass (*n*** **=** **1374)**		
*Crude*	3.1 (2.5–3.9)	**<** **0.001**
*Adjusted all variables from table 1 with p* *≤* *0.05*	2.9 (2.3–3.6)	**<** **0.001**
*Adjusted all variables from table 1 and all variables before ROTEM analysis from table 2*	2.2 (1.7–2.9)	**<** **0.001**
Table 3B Cox proportional hazards models: does fresh frozen plasma have an impact on 30-day mortality?		
***Model***	***HR (95% CI)***	***p‑value***
**All patients (*n*** **=** **2197)**		
*Crude*	1.0 (0.8–1.3)	*0.9*
*Adjusted all variables from table 1 with p* *≤* *0.05*	1.1 (0.9–1.5)	*0.3*
*Adjusted all variables from table 1 and all variables before ROTEM analysis from table 2*	1.1 (0.8–1.4)	*0.6*
**Patients with CT**_**INTEM**_** >** **226s (*n*** **=** **701)**		
*Crude*	0.7 (0.5–0.9)	**0.04**
*Adjusted all variables from table 1 with p* *≤* *0.05*	0.9 (0.6–1.2)	*0.3*
*Adjusted all variables from table 1 and all variables before ROTEM analysis from table 2*	0.8 (0.5–1.1)	*0.1*

Data are expressed as hazard ratios (*HR*) with 95% confidence interval (*CI*). Hazard ratios of CT_INTEM_ (clotting time) in seconds (s) adjusted for confounders. Cox proportional hazards models were used to adjust for confounding factors

*ROTEM* rotational thromboelastometry

^*a*^Sensitivity analyses in randomly split group: The data were randomly split into two groups based on the month they underwent ROTEM analysis in an alternating manner. To avoid any bias associated with the month of ROTEM analysis, we started each year with another group, also alternating

Patients with CT_INTEM_ > 226s had more cardiothoracic and vascular surgery with cardiopulmonary bypass (Table 1). Cardiopulmonary bypass requires anticoagulation, which typically prolongs CT_INTEM_. Therefore, two subgroup analyses were done in patients with and without cardiopulmonary bypass. A CT_INTEM_ > 226s was associated with a significantly increased 30-day mortality only in patients without cardiopulmonary bypass (adjusted HR: 2.2; 95% CI 1.7–2.9, *p* < 0.001; Table 3a; Fig. 2; NNS: 3, PPV: 0.47, NPV: 0.82). In contrast, a CT_INTEM_ > 226s in patients with cardiopulmonary bypass was not significant in the second adjusted analysis (adjusted HR: 1.5; 95% CI 0.9–2.4, *p* = 0.1; Table 3a; NNS: 13, PPV: 0.16, NPV: 0.92).

**Fig. 3:**
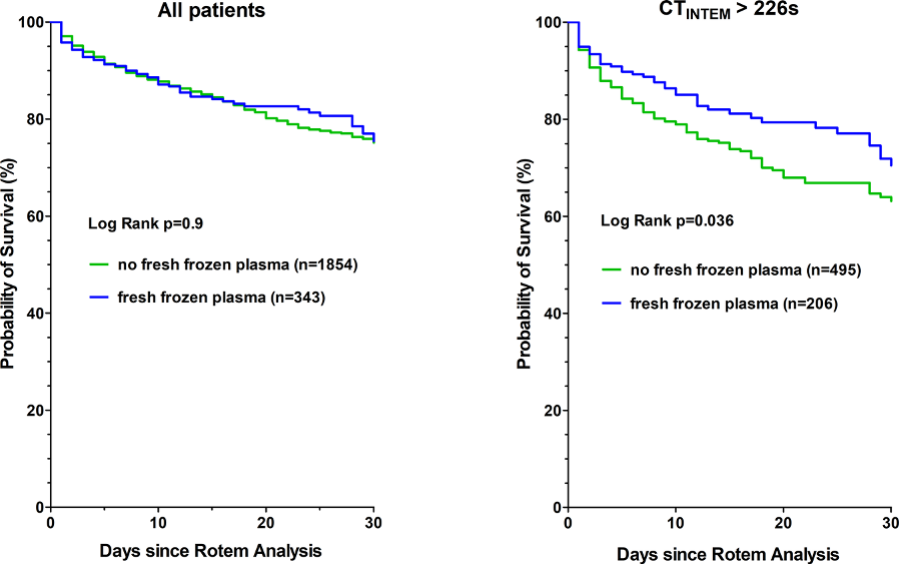
Kaplan-Meier survival plots for treatment with fresh frozen plasma versus no treatment with fresh frozen plasma during 30-day follow-up. The fresh frozen plasma treatment followed after the ROTEM analysis. The left graph includes all patients, the right graph only patients with CT_INTEM_ > 226s. *CT*_*INTEM*_ clotting time, *ROTEM* rotational thromboelastometry

A therapeutic approach for patients with CT_INTEM_ > 226s is the administration of fresh frozen plasma. The 30-day mortality rate seems lower in patients with CT_INTEM_ > 226s treated with fresh frozen plasma than in patients without treatment (Fig. 3). After adjustment for confounding factors, however, the risk reduction is not significant (Table 3b).

## Discussion

Patients with CT_INTEM_ over 226 s had a significantly increased independent risk for 30-day mortality. The overall mortality was 19%. This is consistent with previous reports in trauma patients with bleeding [9, 10]. In our study the definition of bleeding is determined at the discretion of the treating physician. The literature shows no uniform definition for major bleeding [11]. The International Society on Thrombosis and Haemostasis provides a clear definition for nonsurgical patients and a proposed definition for surgical patients, the latter being less objective and partly subjective [12, 13]. While management principles are generally similar, the underlying pathophysiology of major bleeding differs across contexts. Multiple factors contribute to the complex causes of bleeding in trauma and in surgical patients that include blood loss, hemodilution, acquired platelet dysfunction, coagulation factor consumption in extracorporeal circuits, activation of fibrinolytic, fibrinogenolytic and inflammatory pathways and hypothermia [14].

However, early mortality is caused by continued hemorrhage, hemorrhagic shock, trauma-induced coagulopathy and incomplete resuscitation. Numerous factors are known to be associated with a higher complication risk including death after bleeding. These factors include, e.g., age, acute kidney injury, acute lung injury, emergency at hospital admission, bleeding, coagulation disorders and gastrointestinal ischemia [10, 15–19]. Moreover, in patients undergoing cardiopulmonary bypass residual heparin effect or protamine overdose can be one reason for prolongation of CT_INTEM_ [20]. This is consistent with our findings that CT_INTEM_ > 226s were associated with higher 30-day mortality only in patients without cardiopulmonary bypass. There are limited investigations regarding clotting time CT_INTEM_ in the context of bleeding and patient outcome; however, a prolongation of CT_INTEM_ has been observed in nonsurviving trauma patients [4, 5]. Interestingly, we observed the highest difference in nonsurvivors versus survivors, with the exception of a greatly increased need for coagulation factors in the presence of gastrointestinal ischemia (Appendix 2). Gastrointestinal ischemia might be a result of shock during bleeding and a result of the catecholamine treatment in hypovolemic patients and may be a sign for uncontrolled hemorrhage; however, in cardiac surgery gastrointestinal ischemia could be also a potential risk for abnormal hemostatics [21]. To avoid that gastrointestinal ischemia or any other adverse event is a confounder in our analysis, we split the outcome in the time before and after ROTEM analysis (Table 2). The occurrence of all adverse events before ROTEM analysis was adjusted in our sensitive analysis as confounders. This includes also the occurrence of gastrointestinal ischemia. Nevertheless, CT_INTEM_ over 226 s remained an independent risk factor for 30-day mortality (Table 3a).

We speculate that the leading cause of death in patients with CT_INTEM_ over 226 s is a hemostatic dysfunction in the intrinsic pathway. The intrinsic pathway begins with factor XII (Hageman factor XII), which activates factor XI. Patients deficient in factor XII do not exhibit bleeding complications, while factor XI deficient patients (hemophilia C or Rosenthal syndrome) sometimes experience bleeding, suggesting factor XI plays a role in hemostasis independent of the contact pathway [22, 23]; however, the reasons for the prolongation might be highly speculative. Different reasons resulting in a prolonged CT_INTEM_ are heparin and release of endogenous heparinoids from the microcirculation due to glycocalyx shedding [24], heparin-like effect (trauma, cardiopulmonary bypass and liver disease e.g. cirrhosis) [24–28], protamine overdose (results in factor V inhibition) [29], direct oral anticoagulants [30–32], fibrinogen deficiency, factor XII deficiency (consumption due to hyperfibrinolysis) [33], other factor deficiencies in the intrinsic pathway (VIII, IX and XI) or the common pathway (II, V and X).

This abnormal parameter during bleeding requires a differentiated treatment strategy including protamine, tranexamic acid, treatment of platelet dysfunction with desmopressin or platelet concentrates, treatment of factor XIII deficiency, and treatment of factor deficiencies of the extrinsic and intrinsic pathway [2]. Following the recommendations of Weber et al., we performed CT_EXTEM_ to identify and correct extrinsic pathway abnormalities using factor concentrates and fibrinogen. CT_INTEM_ was assessed to address intrinsic pathway deficits, with the addition of fresh frozen plasma considered as advised [2, 6–8, 34].

The use of fresh frozen plasma is controversial. Patients without a coagulopathy, bleeding indication or need for massive transfusion have been shown to experience higher mortality when given fresh frozen plasma. Therefore, careful evaluation of the indications for the use of fresh frozen plasma is vital as inappropriate administration may increase the risk of death [35].

In Europe a coagulation disorder resulting in massive bleeding is generally managed first by the addition of coagulation factor concentrates. Studies have shown that goal-directed coagulation management is associated with a reduced incidence of massive transfusion and lower requirements for red blood cells and fresh frozen plasma [36, 37]. Studies have shown that prothrombin complex concentrate reverses coagulopathy faster and more effectively than fresh frozen plasma, achieves higher clotting factor concentrations with smaller infusion volumes [8, 11, 38, 39]. In contrast, in the USA fresh frozen plasma is often used to maintain the red blood cell-plasma ratio and to replace losses due to bleeding and even serves as a volume expander [40, 41]. Nevertheless, studies have shown a better efficacy when both prothrombin complex concentrate and fresh frozen plasma are used together [42].

This coagulation treatment supports the relevance of other coagulation factors not included in prothrombin complex concentrates and fibrinogen to treat bleeding in massive hemorrhage which might affect 4–6% of patients with trauma-induced coagulopathy [36, 37]. This is consistent with our findings and a previous study that fresh frozen plasma does not increase 30-day mortality during bleeding and coagulation deficits [43]; however, the effects of correcting abnormal clotting with fresh frozen plasma on morbidity and mortality need to be further investigated [36, 44].

The most important limitation of this study is the lack of CT_HEPTEM_ results as the INTEM/HEPTEM CT ratio is the key to differentiate heparin-like effects from factor deficiencies. In the absence of HEPTEM measurements, our interpretation of CT_INTEM_ prolongation remains uncertain as it may be influenced not only by residual heparin or heparin-like substances, but also by endogenous anticoagulants (e.g., increased antithrombin activity), impaired synthesis or consumption of clotting factors, or other coagulopathies. In addition, we did not have systematic data on other relevant laboratory values (e.g., pH, base excess, hemoglobin) or vital parameters, which limits our ability to assess whether patients with prolonged CT_INTEM_ suffered more from severe shock. Another limitation of our study is that although the majority of patients presented with hemorrhagic shock, we cannot fully exclude the contribution of other mechanisms, such as cardiogenic or septic shock, to the observed coagulation changes. Our dataset only included information on adverse events, as listed in Table 2. To address this, we performed a sensitivity analysis by randomly dividing the cohort into two groups to confirm our results. Therefore, while our adjusted analyses and moderate ROC-AUC suggest that CT_INTEM_ has some discriminatory value, the findings should be interpreted with caution and always in conjunction with other clinical information. A larger multicenter study, including HEPTEM is needed to clarify these mechanisms in a more precise population and confirm our findings.

In summary, in our study CT_INTEM_ over 226 s might be an independent risk factor for 30-day mortality in patients with bleeding and could be useful for decision making regarding treatment with fresh frozen plasma.

## Supplementary Information

Appendix 1 and 2

## Data Availability

Data will be made available on reasonable request.
