# Supplementary material for: A clotting time longer than 226 s in the INTEM channel of the thromboelastometer is an independent risk factor for mortality during bleeding
Source: Anaesthesiologie. 2025 Nov 10;74(12):818–26. doi: 10.1007/s00101-025-01602-w (PMC12660322; doi:10.1007/s00101-025-01602-w)
Supplement: Supplementary file 1 — Appendix 1 and 2 [file 101_2025_1602_MOESM1_ESM.docx]

**Supplementary material** for the article "**A clotting time longer than 226 s in the INTEM channel of the thrombelastometer is an independent risk factor for mortality, during bleeding**" by Bomberg H, Görlinger K, Wagenpfeil S et al. (2025) *Die Anaesthesiologie*

The article and supplementary material are available at ww.springermedizin.de. Please enter the article title in the search field.

**Appendix 1: Proof of plausibility**

| ***Items in the registry*** | ***Indications of implausible data*** |
| --- | --- |
| Day of rotational thromboelastometry analysis | - Implausible if rotational thromboelastometry analysis is performed before admission or after discharge from hospital |
| Age | - Implausible if rotational thromboelastometry analysis takes place before date of birth |
| Body mass index | - Implausible if body height and body weight are not consistent (e.g. height: 180 cm, weight: 10 kg) |
| Emergency | - Implausible if the case was not noted as an emergency in the medical records |
| Department | - Implausible if the department cannot be found in the medical records |
| Re-operation after bleeding | - Implausible if the case was not noted as a re-operation after bleeding in the medical records |
| Comorbidities | - Implausible if the comorbidities cannot be found in the medical records |
| Anticoagulation | - Implausible if the anticoagulation cannot be found in the medical records |
| 30-day mortality | - Implausible if the patient was discharged alive after 30 days or is still living in hospital after 30 days |
| Renal replacement therapy | - Implausible if the renal replacement therapy was not found in the medical records |
| Adverse events | - Implausible if the adverse events cannot be found in the medical records |
| All variables in Table 2, except renal replacement therapy, mortality and adverse events | - Implausible if the total number recorded in the medical records does not match the sum of the time points before the rotational thromboelastometry analysis and after the rotational thromboelastometry analysis (e.g. the total number of administrated packed red blood cells during the hospital stay = administrated packed red blood cells before the rotational thromboelastometry analysis **plus** administrated packed red blood cells after the rotational thromboelastometry analysis) |

**Appendix 2 Survivor versus non-survivor in patients with CT_INTEM_ (Clotting Time) > 226 seconds (s)**.
 Continuous variables are expressed as mean and standard deviation. Categorical variables are presented as numbers (percentages). ICU (Intensive care unit). Rotem (Rotational thromboelastometry). Packed red blood cells (450mL), Platelet concentrate (270mL), Fresh frozen plasma (300mL).

|  | | | | | |  |
| --- | --- | --- | --- | --- | --- | --- |
| ***30-day mortality in patients with CT_INTEM_ > 226s*** | | | | | |  |
|  |  | | | | |  |
|  | ***survivor*** | | ***non-survivor*** | | ***p-value*** |  |
|  | ***(n=501)*** | | ***(n=200)*** | |  |  |
|  |  |  |  |  |  |  |
| *Mech. ventilated (h)* | 75 | ±201 | 45 | ±97 | ***0.04*** |  |
| *Renal replacement therapy (%)* | 109 | (22) | 104 | (52) | ***<0.001*** |  |
| ***Length of stay (days)*** |  |  |  |  |  |  |
| - *In ICU* | 13 | ±24 | 6 | ±7 | ***<0.001*** |  |
| - *In hospital* | 25 | ±34 | 7 | ±8 | ***<0.001*** |  |
| *30-day mortality (%)* | **─** | | 200 | (100) | **─** |  |
| *Packed red blood cells (n)* | 4 | ±7 | 4 | ±5 | *0.6* |  |
| *Platelet concentrate (n)* | 2 | ±2 | 2 | ±2 | *0.5* |  |
| *Fresh frozen plasma (n)* | 2 | ±9 | 2 | ±9 | *0.9* |  |
| *Prothrombin concentrate (IU)* | 800 | ±1590 | 1283 | ±2264 | ***0.001*** |  |
| *Fibrinogen (g)* | 0.8 | ±2 | 1.6 | ±3 | ***<0.001*** |  |
| *Recombinant Factor VIIa (IU)* | 16 | ±177 | 4 | ±37 | *0.3* |  |
| *Antithrombin III (IU)* | 124 | ±1060 | 112 | ±915 | *0.6* |  |
| *Factor XIII (IU)* | 27 | ±200 | 50 | ±303 | *0.2* |  |
| ***Adverse events*** |  |  |  |  |  |  |
| - *Pneumonia (%)* | 79 | (16) | 19 | (10) | ***0.03*** |  |
| - *Pulmonary embolism (%)* | 5 | (1) | 2 | (1) | *1* |  |
| - *Acute myocardial infarction (%)* | 1 | (0.2) | 4 | (2) | ***0.03*** |  |
| - *Embolic apoplexy (%)* | 14 | (3) | 7 | (4) | *0.6* |  |
| - *Peripheral arterial embolism and thrombosis (%)* | 6 | (1) | 3 | (2) | *0.7* |  |
| - *Gastrointestinal ischemia (%)* | 14 | (3) | 21 | (11) | ***<0.001*** |  |
| - *Gastrointestinal bleeding (%)* | 3 | (1) | 5 | (3) | ***0.05*** |  |
